# A systematic review of the international published literature relating to quality of institutional care for people with longer term mental health problems

**DOI:** 10.1186/1471-244X-9-55

**Published:** 2009-09-07

**Authors:** Tatiana L Taylor, Helen Killaspy, Christine Wright, Penny Turton, Sarah White, Thomas W Kallert, Mirjam Schuster, Jorge A Cervilla, Paulette Brangier, Jiri Raboch, Lucie Kališová, Georgi Onchev, Hristo Dimitrov, Roberto Mezzina, Kinou Wolf, Durk Wiersma, Ellen Visser, Andrzej Kiejna, Patryk Piotrowski, Dimitri Ploumpidis, Fragiskos Gonidakis, José Caldas-de-Almeida, Graça Cardoso, Michael B King

**Affiliations:** 1 Research Department of Mental Health Sciences, UCL Medical School, London, UK; 2 Division of Mental Health, St. George's University London, London, UK; 3 Department of Psychiatry and Psychotherapy, University Hospital Carl Gustav Carus, Technische Universitaet Dresden, Dresden, Germany; 4 CIBERSAM, Universidad de Granada, Granada, Spain; 5 Psychiatric Department of the First Faculty of Medicine, Charles University, Prague, Czech Republic; 6 Department of Psychiatry, Medical University Sofia, Sofia, Bulgaria; 7 Dipartimento di Salute Mentale, University of Trieste, Trieste, Italy; 8 Psychiatry, University Medical Centre Groningen, University of Groningen, Groningen, Netherlands; 9 Department of Psychiatry, Wroclaw Medical University, Wroclaw, Poland; 10 University Mental Health Research Institute (UMHRI), Athens, Greece; 11 Department of Mental Health, Faculdade de Ciencias Medicas, New University of Lisbon, Lisbon, Portugal

## Abstract

**Background:**

A proportion of people with mental health problems require longer term care in a psychiatric or social care institution. However, there are no internationally agreed quality standards for institutional care and no method to assess common care standards across countries.

We aimed to identify the key components of institutional care for people with longer term mental health problems and the effectiveness of these components.

**Methods:**

We undertook a systematic review of the literature using comprehensive search terms in 11 electronic databases and identified 12,182 titles. We viewed 550 abstracts, reviewed 223 papers and included 110 of these. A "critical interpretative synthesis" of the evidence was used to identify domains of institutional care that are key to service users' recovery.

**Results:**

We identified eight domains of institutional care that were key to service users' recovery: living conditions; interventions for schizophrenia; physical health; restraint and seclusion; staff training and support; therapeutic relationship; autonomy and service user involvement; and clinical governance. Evidence was strongest for specific interventions for the treatment of schizophrenia (family psychoeducation, cognitive behavioural therapy (CBT) and vocational rehabilitation).

**Conclusion:**

Institutions should, ideally, be community based, operate a flexible regime, maintain a low density of residents and maximise residents' privacy. For service users with a diagnosis of schizophrenia, specific interventions (CBT, family interventions involving psychoeducation, and supported employment) should be provided through integrated programmes. Restraint and seclusion should be avoided wherever possible and staff should have adequate training in de-escalation techniques. Regular staff supervision should be provided and this should support service user involvement in decision making and positive therapeutic relationships between staff and service users. There should be clear lines of clinical governance that ensure adherence to evidence-based guidelines and attention should be paid to service users' physical health through regular screening.

## Background

A proportion of people with mental health problems require longer term care in a psychiatric or social care institution based in hospital or the community. The majority of these people have a diagnosis of schizophrenia [1]. They are also likely to have other problems which have complicated their recovery such as treatment resistance [2], cognitive impairment [3-6]; pre-morbid learning disability [7], substance misuse and other challenging behaviours [3,8]. Their illness impacts on their capacity to make informed choices for themselves and to actively participate in their care, putting them at risk of exploitation and abuse from others, including those who care for them. To combat this and ensure institutions are providing appropriate treatment and care, many countries have set up their own systems for monitoring the care provided. However, there are no internationally agreed quality standards for institutional care and no method to assess common care standards across countries.

The DEMoBinc (Development of a European Measure of Best Practice for People with Long Term Mental Illness in Institutional Care) Study is a collaboration between eleven centres in ten European countries. It aims to build and test an international toolkit that can reliably assess the care and living conditions of adults with longer term mental health problems whose levels of need necessitate their living in psychiatric or social care institutions [9]. In order for the toolkit to have cross-country validity, it was recognised that it needed to incorporate core characteristics of care, whatever their service context. Therefore, an emphasis on the Recovery Model [10] has been included from the early stages of development since it incorporates key aspects of mental health promotion that are agreed internationally, such as advocating non-coercive relationships between professionals and service users, empowerment, patient autonomy and facilitation of increasing levels of independence. The initial stages of development of the toolkit comprised a literature review of aspects of institutional care associated with service users' recovery and an international Delphi exercise investigating key stakeholders' views of the "critical success factors" involved in promoting service users' recovery in these settings [11]. This paper reports on the findings of the literature review.

The scope of the literature review was necessarily broad since we wanted to include all core components of institutional care. Our review was carried out systematically but also has a narrative component whereby we synthesised the best available evidence in this field to identify areas (or "domains") of care and components of these domains for inclusion in the toolkit. Conventional systematic reviews are often unable to provide a critical analysis of a complex body of literature. This is particularly the case in assessing evidence on the components of care that constitute an "ideal" institution. Thus, we adopted the approach which has been described as a 'critical interpretative synthesis' [12] which allows for the analysis of a body of literature which is "large, diverse and complex" and includes both quantitative and qualitative methodologies. Instead of analysing the literature using pre-determined outcomes, key concepts are defined after the synthesis of the findings, allowing for greater exploration of a broad array of outcomes and experiences.

### Aims

We undertook a systematic review of the international literature published in peer reviewed journals since 1980 with the aims of:

1. identifying key components of institutional care for people with longer term mental health problems.

2. evaluating the effectiveness of these components.

3. undertaking a critical interpretative synthesis of the evidence in order to identify the domains of institutional care that are key to service users' recovery.

## Method

### Eligibility

#### Inclusion criteria

We included papers that examined factors associated with quality of care, of adults of working age with longer term mental health problems living in institutional care in hospital or the community. Papers that examined the relationship between quality of care and operational systems, staffing, staff training, supervision and support were included as well as papers that investigated living conditions and those that investigated specific approaches to improve the quality of care. The review was limited to papers published since 1980 since much of the deinstitutionalisation across Europe has taken place in the last 30 years.

#### Exclusion criteria

Papers were excluded if the focus was irrelevant to the aims of our systematic review due to one or more of the following:

A) the results were specific to a client group that did not meet our inclusion criteria (e.g. child or adolescent patients; patients in prison; patients with mental illnesses unlikely to require long-term institutional care; patients with dementia; patients with primary drug or alcohol problems) and could not be extrapolated to adults of working age with long term mental health problems living in institutional care in hospital or in the community;

B) the study was carried out in unrelated settings (e.g. short-term wards or specialist units not focusing on patients with long-term mental health problems or patients living at home or in non-institutional community settings);

C) the results reported were confined to an exceptional setting, culture, client group or intervention and could not be extrapolated internationally (e.g. national mental health legislation or a very specific service context);

D) studies that examined patients' quality of life or satisfaction in isolation from their context in institutional care, or whose focus was too broad for its results to be useful for the aims of this systematic review.

E) studies that reported on drug trials.

Where a systematic review was included, we did not examine each paper contained within it. Nor did we include editorials, letters, books or book chapters.

### Search strategy

#### Search terms

The following terms were used to identify relevant articles: mental patient*; mental* ill*; mental disease*; mental* deficien*; mental disorder*; schizophreni*; mental* disab*; mental* retard*; psycho*; severe mental illness; psychiatr*; mental health patient; delivery; standard*; quality; benchmark*; evaluat* near care; evaluat* near health care; guideline*; quality of life; treatment satisfaction; model; evaluation stud*; patient* satisfaction; clinical guideline*; evidence based medicine; psychiatric rehabilitation; rehabilitat*; activities of daily living; art therapy; bibliotherapy; dance therapy; exercise therapy; music therapy; occupational therapy; rehabilitation, vocation*; physical restrain*; hold* down; clinical hold*; human right*; patient right*; behaviour control; collaboration; recovery; empowerment; consumer movement; mental health care; mental health cent*; mental hospital*; psychiatric department*; community mental health; community mental health cent*; community psychiatric nurs*; mental health service*; hospital*; inpatient*; institut* care; institution*; deinstitution*; social work, psychiatric; managed care; community mental health care; architectural accessibility; elevator* and escalator*; floor* and floorcovering*; interior design and furnishing*; location directorie* and sign*; parking facilit*; health facility environment; patient* room*; rehabilitation center*; sheltered workshop*; residential facility*; assisted living facility*; group home*; halfway house*; homes for the aged; nursing home*; nursing care; nursing services; rehabilitation; activities of daily living; rehabilitation, vocational; self care.

All search terms were adapted for each database.

The following electronic databases were searched:

Medline: 1980 - May 2007

Embase: 1980 - May 2007

PsycINFO: 1980 - May 2007

CINAHL: 1982 - May 2007

The Cochrane Library as of Issue 2, 2007

Web of Knowledge: 1980 - June 2007

ASSIA: 1980 - July 2007

International Bibliography of the Social Sciences: 1980 - June 2007

Sociological Abstracts: 1980 - July 2007

Social Science Citation Index: 24 October 2007

Science Citation Index EXPANDED: 24 October 2007

Author or paper searches were clarified, where necessary, using Google scholar. First authors of included articles were contacted for additional published or unpublished material when appropriate. Principal investigators from each of the countries participating in the DEMoBinc study provided references or copies of relevant papers that had not been identified from the databases listed above. No relevant studies were found which had been missed by our search.

### Selection of articles

TT and HK screened all relevant abstracts identified in the searches for eligibility. TT, HK, MK, CW, PT, and SW reviewed a draft list of articles for possible inclusion and a final list was agreed by consensus.

### Assessment of methodological quality

**Figure 1:**
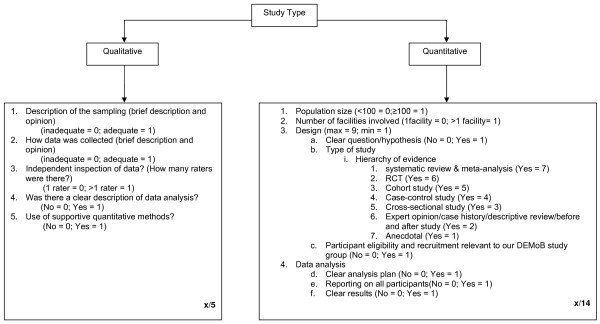
**Quality assessment instructions (separate file)**.

The quality of papers was rated, by consensus, by TT and HK using the criteria shown in Figure 1. Separate criteria were used for qualitative and quantitative research papers. These criteria were derived from recommended approaches [13-16] and additional items specific to this review. Quantitative papers were assessed on: (1) population size; (2) number of facilities from which participants were recruited; (3) design, (which included clarity of the research question or hypothesis, the type of methodology used [16] and relevance of the participants to the aims of the review); (4) data analysis (which included clarity of the analysis plan, reporting on all participants and clarity of the results). These criteria provided a maximum score of 14 points. Qualitative papers were assessed on: (1) sampling; (2) data collection; (3) data inspection; (4) data analysis; (5) the use of supportive quantitative methods. These criteria provided a maximum score of five points. Where a paper included both types of research two separate quality assessments were carried out.

### Data extraction and management

Data on authors, year of publication, study setting, study design, population, study focus, assessment measures used and outcomes were extracted by TT. Results were extracted and compiled in summary form.

Included papers were grouped by theme and domains were determined once all data were compiled. TT, HK, MK, CW, PT, and SW agreed the domains by consensus. Allocation of papers to domains was carried out by TT, while HK categorised a randomly selected sample of 20 of the included papers to ensure reliability. Nineteen of the 20 papers were matched. Efficacy data (e.g. effect size, number needed to treat [NNT], risk ratio [RR]), *P*-value and 95% confidence intervals from meta-analyses and randomised controlled trials (RCTs) were reported if provided within the paper or if calculations could be performed using the data provided by the authors. The National Institute for Clinical Excellence (NICE) in the UK considers that an effect size of 0.20 to 0.49 is small, 0.50 to 0.79 is medium and 0.80 or over is large. We have used this guide in the text when reporting effect sizes. Findings are summarised in the text for each domain. More weight was given to papers of higher quality and findings supported by multiple studies.

## Results

**Figure 2:**
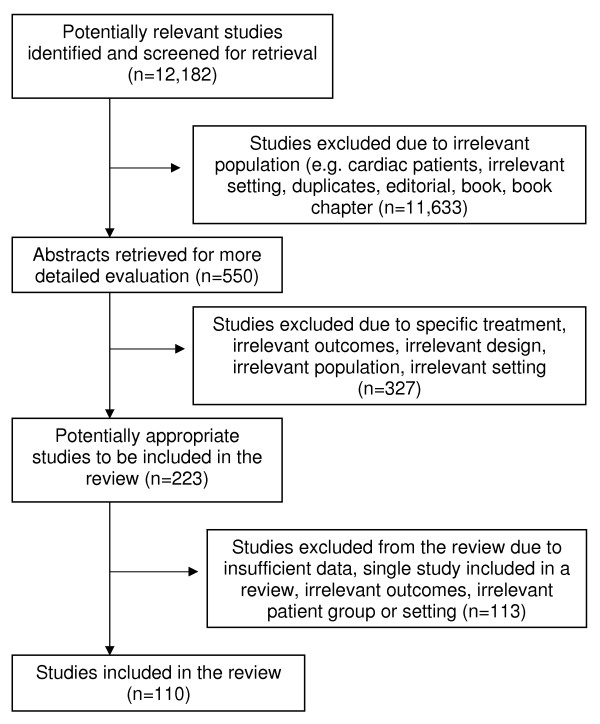
**Study flow diagram (separate file)**.

A total of 12,182 relevant articles were identified through the search strategy (see Figure 2). After further inspection of abstracts and papers, 12,073 articles were excluded due to duplications or exclusion criteria (see Additional file 1). One hundred and ten articles were included in the review.

### Study Characteristics

Papers were grouped into at least one of eight domains: living conditions; interventions for schizophrenia; physical health; restraint and seclusion; staff training and support; therapeutic relationship; service user involvement and autonomy; and clinical governance.

The main characteristics of papers included within each domain are shown in Tables 1, 2, 3, 4, 5, 6, 7, 8, 9, 10, 11, 12, 13, 14, 15, 16, 17, 18, 19 and 20. Included papers came from 19 countries and were published between 1980 and 2007. The majority came from the USA (46 papers) and the UK (27 papers). Five were international multicentre studies [17-21]. Fifty-six studies specifically included patients with schizophrenia but many did not describe participants' diagnoses. The types of facilities investigated included both hospital-based (e.g. wards) and community-based (e.g. boarding homes, nursing homes, supported housing) institutions. Several studies did not describe the specific type of facility and some studies included outpatient and inpatient services.

**Table 1:** Characteristics and quality of studies included in living conditions domain

**Study (Country)**	**Type(s) of Mental Illness**	**Number of Participants**	**Type of Study** **(Type of Setting)**	**Quality Assessment**
Baker & Douglas 1990 (USA)	Mostly schizophrenia	729	Cohort study (supported and unsupported community housing)	10/14

Brunt & Hansson 2002 (Sweden)	Severe mental illness	33 patients 50 staff	Cross-sectional study (small group homes)	8/14

Corrigan 1990 (USA)	Severe mental illness	Not applicable	Descriptive review (hospital ward and outpatient settings)	6/14

Cournos 1987 (USA)	Chronic mental illness	Not specified	Descriptive review (community residential settings)	6/14

Cullen et al. 1997 (UK)	Not specified	42	Cross-sectional study (hospital and community residential settings)	7/14

Dijkstra et al. 2006 (The Netherlands)	Not confined to mental health patients	5412	Systematic review (30 studies) (hospital-based settings)	12/14

Fakhoury et al. 2002 (UK)	Severe and enduring mental illness	3,577 patients 166 staff	Systematic review (28 studies) (supported housing)	7/14

Fakhoury et al. 2005 (UK)	Schizophrenia or related psychotic disorder	41 patients 39 staff	Cross-sectional study (supported housing)	5/5; 9/14

Hawthorne et al. 1994 (USA)	Severe mental illness	104	Before and after study (community-based residential settings)	9/14

Johansson & Eklund 2004 (Sweden)	Minority schizophrenia	61	Cross-sectional study (psychiatric inpatient ward)	8/14

Kruzich & Kruzich 1985 (USA)	Majority schizophrenia	87	Cross-sectional study (residential care settings)	10/14

Lehman et al. 2004 (USA)	Schizophrenia	Not applicable	Clinical guidance (inpatient and outpatient settings)	Not applicable

Mares et al. 2002 (USA)	Severe mental illness	164	Cross-sectional study (board and care homes)	9/14

Rickard et al. 2002 (Spain and UK)	Functional psychotic illness	136	Cross-sectional study (community residences)	10/14

Santone et al. 2005 (Italy)	Severely impaired patients	265 facilities	Cross-sectional study (residential setting)	9/14

Shrivastava et al. 1999 (UK)	Not specified	Not specified	Descriptive review (psychiatric unit)	6/14

Trauer et al. 2001 (Australia)	Not specified	125	Cohort study (community care unit)	10/14

van Wel et al. 2003 (The Netherlands)	Not specified	129	Cross-sectional study (psychiatric hospital)	8/14

**Table 2:** Characteristics and quality of studies included in interventions domain: Cognitive behavioural therapy

**Study (Country)**	**Type(s) of Mental Illness**	**Number of Participants**	**Type of Study** **(Type of Setting)**	**Quality Assessment**
Barrowclough et al. 2006 (UK)	Schizophrenia or schizoaffective disorder	113	RCT (inpatient and outpatient settings)	12/14

Lehman et al. 2004 (USA)	Schizophrenia	Not applicable	Clinical guidance (inpatient and outpatient settings)	Not applicable

NICE 2002 (UK)	Schizophrenia	Not applicable	Clinical guidance (inpatient and outpatient settings)	Not applicable

Pfammatter et al. 2006 (Switzerland)	Schizophrenia or psychosis	Not specified	Systematic review & meta-analysis (4 meta-analyses, 17 studies) (setting not specified)	14/14

Pilling et al. 2002b (UK)	Schizophrenia or related disorder	528	Systematic review & meta-analysis (8 studies) (setting not specified)	14/14

Turkington et al. 2006 (UK)	Schizophrenia	336	RCT (inpatient and outpatient settings)	12/14

**Table 3:** Characteristics and quality of studies included in interventions domain: Family interventions and psychoeducation

**Study (Country)**	**Type(s) of Mental Illness**	**Number of Participants**	**Type of Study** **(Type of Setting)**	**Quality Assessment**
Carrà et al. 2007 (Italy)	Schizophrenia	101 relatives	RCT (setting not specified)	12/14

Lehman et al. 2004 (USA)	Schizophrenia	Not applicable	Clinical guidance (inpatient and outpatient settings)	Not applicable

McFarlane et al. 2003 (USA)	Schizophrenia	Not specified	Descriptive review (setting not specified)	6/14

Mueser & Bond 2000 (USA)	Schizophrenia	Not specified	Descriptive review (inpatient and outpatient settings)	6/14

NICE 2002 (UK)	Schizophrenia	Not applicable	Clinical guidance (inpatient and outpatient settings)	Not applicable

Pekkala & Merinder 2002 (Finland)	Schizophrenia or related serious mental illness	1125	Systematic review & meta-analysis (10 studies) (inpatient and outpatient settings)	14/14

Pfammatter et al. 2006 (Switzerland)	Schizophrenia or psychosis	Not specified	Systematic review & meta-analysis (31 studies) (setting not specified)	14/14

Pharoah et al. 2006 (UK)	Schizophrenia or schizophrenia-like conditions	4444	Systematic review & meta-analysis (43 studies) (community settings)	14/14

Pilling et al. 2002b (UK)	Schizophrenia or related disorder	1128	Systematic review & meta-analysis (18 studies) (setting not specified)	14/14

Pitschel-Walz et al. 2006 (Germany)	Schizophrenia or schizoaffective disorder	236 patients 125 relatives	RCT (hospital wards)	12/14

Rabovsky & Stoppe 2006 (Germany)	Schizophrenia	Not applicable	Discussion paper (inpatient setting)	7/14

Rummel-Kluge et al. 2006 (Germany, Austria, Switzerland)	Any, but focuses on patients with schizophrenia	337 facilities	Cross-sectional study (psychiatric institutions)	10/14

**Table 4:** Characteristics and quality of studies included in interventions domain: Vocational therapy

**Study (Country)**	**Type(s) of Mental Illness**	**Number of Participants**	**Type of Study** **(Type of Setting)**	**Quality Assessment**
Bond et al. 1997 (USA)	Severe mental illness	2191	Systematic review (17 studies) (setting not specified)	12/14

Bond et al. 2001 (USA)	Severe mental illness	Not applicable	Descriptive review (setting not specified)	6/14

Crowther et al. 2001 (USA)	Schizophrenia and schizophrenia-like disorders, bipolar disorder, depression with psychotic features	2539	Systematic review & meta-analysis (18 studies) (inpatient and outpatient settings)	14/14

Drake et al. 2003 (USA)	Not specified	499	Cohort study (setting not specified)	9/14

Lehman et al. 2004 (USA)	Schizophrenia	Not applicable	Clinical guidance (inpatient and outpatient settings)	Not applicable

Mueser & Bond 2000 (USA)	Schizophrenia	Not specified	Descriptive review (inpatient and outpatient settings)	6/14

NICE 2002 (UK)	Schizophrenia	Not applicable	Clinical guidance (inpatient and outpatient settings)	Not applicable

Twamley et al. 2003 (USA)	Schizophrenia and other disorders	1617	Systematic review & meta-analysis (11 studies) (setting not specified)	13/14

**Table 5:** Characteristics and quality of studies included in interventions domain: Social skills training

**Study (Country)**	**Type(s) of Mental Illness**	**Number of Participants**	**Type of Study** **(Type of Setting)**	**Quality Assessment**
Bustillo et al. 2001 (USA)	Schizophrenia, severe mental illness	962	Systematic review (5 studies) (setting not specified)	12/14

Lehman et al. 2004 (USA)	Schizophrenia	Not applicable	Clinical guidance (inpatient and outpatient settings)	Not applicable

NICE 2002 (UK)	Schizophrenia	Not applicable	Clinical guidance (inpatient and outpatient settings)	Not applicable

Pfammatter et al. 2006 (Switzerland)	Schizophrenia or psychosis	Not specified	Systematic review & meta-analysis (19 studies) (setting not specified)	14/14

Pilling et al. 2002a (UK)	Schizophrenia or related disorder	417	Systematic review & meta-analysis (9 studies) (setting not specified)	14/14

Roder et al. 2001 (Switzerland, Austria, and Germany)	Schizophrenia	73	Case-control study (psychiatric institution)	8/14

Roder et al. 2002 (Switzerland, Austria, and Germany)	Schizophrenia	105	Case-control study (psychiatric institution)	10/14

**Table 6:** Characteristics and quality of studies included in interventions domain: Cognitive remediation

**Study (Country)**	**Type(s) of Mental Illness**	**Number of Participants**	**Type of Study** **(Type of Setting)**	**Quality Assessment**
Pfammatter et al. 2006 (Switzerland)	Schizophrenia or psychosis	Not specified	Systematic review & meta-analysis (6 meta-analyses, 19 studies) (setting not specified)	14/14

Pilling et al. 2002a (UK)	Schizophrenia or related disorder	203	Systematic review & meta-analysis (5 studies) (setting not specified)	14/14

Wykes et al. 2007 (UK)	Schizophrenia	85	RCT (setting not specified)	11/14

**Table 7:** Characteristics and quality of studies included in interventions domain: Arts therapies

**Study (Country)**	**Type(s) of Mental Illness**	**Number of Participants**	**Type of Study** **(Type of Setting)**	**Quality Assessment**
Gold et al. 2005 (Norway)	Schizophrenia or related psychoses	266	Systematic review & meta-analysis (4 studies) (inpatient settings)	14/14

Ruddy & Milnes 2005 (UK)	Schizophrenia	137	Systematic review & meta-analysis (2 studies) (setting not specified)	14/14

Ruddy & Dent-Brown 2007 (UK)	Schizophrenia	210	Systematic review & meta-analysis (5 studies) (inpatient settings)	14/14

**Table 8:** Characteristics and quality of studies included in interventions domain: Integrated therapy

**Study (Country)**	**Type(s) of Mental Illness**	**Number of Participants**	**Type of Study** **(Type of Setting)**	**Quality Assessment**
Lenroot et al. 2003 (USA)	Schizophrenia	Not applicable	Descriptive review (setting not specified)	5/14

Mueser et al. 2006 (USA)	Schizophrenia or major mood disorder	32	Cohort study (non-residential community settings)	10/14

Roder et al. 2006 (Switzerland)	Schizophrenia	1393	Systematic review (30 studies) (psychiatric institutions)	14/14

**Table 9:** Characteristics and quality of studies included in interventions domain: Treatment of comorbid substance misuse

**Study (Country)**	**Type(s) of Mental Illness**	**Number of Participants**	**Type of Study** **(Type of Setting)**	**Quality Assessment**
Drake et al. 2004 (USA)	Severe mental illness and co-occurring substance use disorder	4,313 residents 1,982 outpatients	Descriptive review (outpatient and inpatient settings)	7/14

Lehman et al. 2004 (USA)	Schizophrenia	Not applicable	Clinical guidance (inpatient and outpatient settings)	Not applicable

Ziedonis et al. 2005 (USA)	Schizophrenia and substance abuse disorder	Not applicable	Clinical guidance (setting not specified)	Not applicable

**Table 10:** Characteristics and quality of studies included in interventions domain: Medication management

**Study (Country)**	**Type(s) of Mental Illness**	**Number of Participants**	**Type of Study** **(Type of Setting)**	**Quality Assessment**
Lehman et al. 2004 (USA)	Schizophrenia	Not applicable	Clinical guidance (inpatient and outpatient settings)	Not applicable

NICE 2002 (UK)	Schizophrenia	Not applicable	Clinical guidance (inpatient and outpatient settings)	Not applicable

**Table 11:** Characteristics and quality of studies included in interventions domain: Compliance therapy

**Study (Country)**	**Type(s) of Mental Illness**	**Number of Participants**	**Type of Study** **(Type of Setting)**	**Quality Assessment**
Eckman et al. 1990 (USA)	Schizophrenia	160 patients unknown number of staff	Case-control study (inpatient, outpatient and community residential settings)	10/14

Eckman et al. 1992 (USA)	Schizophrenia	41	RCT (inpatient and outpatient settings)	11/14

Kemp et al. 1998 (UK)	Majority schizophrenia	74	RCT (inpatient setting)	10/14

Kuipers et al. 1994 (USA)	Chronically mental illness	60	RCT (hospital setting)	10/14

McIntosh et al. 2006 (UK)	Schizophrenia or related severe mental disorders	56	Systematic review & meta-analysis (1 study) (setting not specified)	12/14

Seltzer et al. 1980 (Canada)	Majority schizophrenia	67	RCT (psychiatric institute)	9/14

Streicker et al. 1986 (USA)	Majority schizophrenia	75	Case-control study (psychosocial rehabilitation agency)	9/14

**Table 12:** Characteristics and quality of studies included in interventions domain: Occupational therapy

**Study (Country)**	**Type(s) of Mental Illness**	**Number of Participants**	**Type of Study** **(Type of Setting)**	**Quality Assessment**
Buchain et al. 2003 (Brazil)	Schizophrenia (treatment resistant)	26	RCT (setting not specified)	9/14

Oka et al. 2004 (Japan)	Schizophrenia	52	Before and after study (inpatient and outpatient settings)	9/14

**Table 13:** Characteristics and quality of studies included in interventions domain: Supportive therapy

**Study (Country)**	**Type(s) of Mental Illness**	**Number of Participants**	**Type of Study** **(Type of Setting)**	**Quality Assessment**
Buckley et al. 2007 (UK)	Schizophrenia	1762	Systematic review & meta-analysis (21 studies) (inpatient and outpatient settings)	13/14

**Table 14:** Characteristics and quality of studies included in interventions domain: Coping skills training

**Study (Country)**	**Type(s) of Mental Illness**	**Number of Participants**	**Type of Study** **(Type of Setting)**	**Quality Assessment**
Leclerc et al. 2000 (Canada)	Schizophrenia	99	RCT (inpatient wards and outpatient clinics)	12/14

Lecomte et al. 1999 (Canada)	Schizophrenia	95	RCT (long-stay wards, short-stay wards, outpatient clinic)	11/14

**Table 15:** Characteristics and quality of studies included in physical health domain

**Study (Country)**	**Type(s) of Mental Illness**	**Number of Participants**	**Type of Study** **(Type of Setting)**	**Quality Assessment**
Anath et al. 1992 (USA)	Mostly schizophrenia	75	Cross-sectional study (inpatient setting)	7/14

Bazemore et al. 2005 (USA)	Not specified	102 hospitals	Cross-sectional study (hospital setting)	10/14

Kilian et al. 2006 (Germany)	Schizophrenia, bipolar disorder, major depressive disorder, neurotic disorder, somatoform disorder	363	Cross-sectional study (inpatient setting)	9/14

Lehman et al. 2004 (USA)	Schizophrenia	Not applicable	Clinical guidance (inpatient and outpatient settings)	Not applicable

Mitchell & Malone 2006 (UK)	Schizophrenia	Not specified	Descriptive Review (setting not specified)	6/14

NICE 2002 (UK)	Schizophrenia	Not applicable	Clinical guidance (inpatient and outpatient settings)	Not applicable

Osborn et al. 2003 (UK)	Schizophrenia, schizoaffective disorder or other non-affective chronic psychotic illness	495	Cross-sectional study (general practices)	9/14

Osborn et al 2006 (UK)	Schizophrenia, schizoaffective disorder or other non-affective chronic psychotic illness	222	Cross-sectional study (general practices)	10/14

Tang et al. 2004 (China)	Majority schizophrenia	98	Cross-sectional study (psychiatric rehabilitation facility)	8/14

**Table 16:** Characteristics and quality of studies included in restraint and seclusion domain

**Study (Country)**	**Type(s) of Mental Illness**	**Number of Participants**	**Type of Study** **(Type of Setting)**	**Quality Assessment**
Addington et al. 2005 (Canada)	Schizophrenia	Not applicable	Clinical guidance (inpatient and outpatient settings)	Not applicable

Bower et al. 2000 (USA)	Not specified	Not specified	Systematic review (223 studies) (inpatient psychiatric settings)	7/14

Donat 2002 (USA)	Severe mental illness	53	Case-control study (psychiatric hospital)	6/14

Donat 2003 (USA)	Severe mental illness	53	Case-control study (psychiatric hospital)	7/14

Fisher 1994 (USA)	Not specified	Not applicable	Descriptive review (inpatient settings)	6/14

Gaskin et al. 2007 (Australia)	Not specified	Not specified	Systematic review (16 studies) (psychiatric facilities)	11/14

Janssen et al. 2007 (The Netherlands)	Not specified	Not specified	Cross-sectional study (admission and long-stay psychiatric wards)	10/14

Khadivi et al. 2004 (USA)	Not specified	Not specified	Cross-sectional study (psychiatric inpatient setting)	6/14

Kostecka & Zardecka 1999 (Poland)	Not specified	866	Cross-sectional study (psychiatric hospital wards)	10/14

Lehman et al. 2004 (USA)	Schizophrenia	Not applicable	Clinical guidance (inpatient and outpatient settings)	Not applicable

McCue et al. 2004 (USA)	Not specified	10,753	Cohort study (inpatient setting)	12/14

McGorry et al. 2005 (New Zealand and Australia)	Schizophrenia and related disorders	Not applicable	Clinical guidance (inpatient and outpatient settings)	Not applicable

Muralidharan & Fenton 2006 (USA)	Not specified	0	Systematic review (0 studies) (inpatient setting)	12/14

NICE 2002 (UK)	Schizophrenia	Not applicable	Clinical guidance (inpatient and outpatient settings)	Not applicable

Nelstrop et al. 2006 (UK)	Not specified	Not applicable	Systematic review (36 studies) (inpatient settings)	12/14

Palazzolo et al. 2001 (France)	Not specified	Not specified	Descriptive review (psychiatric hospitals)	6/14

Wynn 2002 (Norway)	Not specified	235	Cross-sectional study (psychiatric hospital)	9/14

Wynn 2004 (Norway)	Majority schizophrenia	12	Qualitative study (hospital wards)	3/5

**Table 17:** Characteristics and quality of studies included in therapeutic relationship domain

**Study (Country)**	**Type(s) of Mental Illness**	**Number of Participants**	**Type of Study** **(Type of Setting)**	**Quality Assessment**
Allen et al 1985 (USA)	Chronic and severe psychiatric disturbances	37	Cross-sectional study (long-term hospital unit)	9/14

Berger 2006 (Canada)	Not specified	46 patients 17 staff	Cross-sectional study (inpatient and outpatient settings)	5/14

Catty 2004 (UK)	Not specified	Not specified	Descriptive review (setting not specified)	6/14

Clarkin et al. 1987 (USA)	Schizophrenia, personality disorder, affective disorder, acute illness	96	Cross-sectional study (inpatient setting)	7/14

Fakhoury et al. 2005 (UK)	Schizophrenia or related psychotic disorder	41 patients 39 staff	Cross-sectional study (supported housing)	5/5; 9/14

Gehrs & Goering 1994 (Canada)	Schizophrenia or schizoaffective disorder	22 client-therapist dyads	Case-control study (continuing care)	9/14

Gigantesco et al. 2002 (Italy)	Not specified	855 patients 265 relatives	Cross-sectional study (inpatient and outpatient settings)	9/14

Hellzén 2004 (Sweden)	Long-term mental illness	32	Focus group (psychiatric group dwellings)	4/5

Howgego et al. 2003 (USA)	Not specified	533 patients 131 case managers/therapists	Systematic review & meta-analysis (2 meta-analyses & 7 studies) (inpatient and outpatient settings)	12/14

Johansson & Eklund 2004 (Sweden)	Minority schizophrenia	61	Cross-sectional study (psychiatric inpatient ward)	8/14

McCabe et al. 1999 (UK)	Schizophrenia	258	Cohort study (psychiatric hospital)	9/14

McCabe & Priebe 2004 (UK)	Severe mental illness	2055	Descriptive review (setting not specified)	7/14

Mueser et al. 2002 (USA)	Serious mental illness	3,079	Descriptive review (inpatient and outpatient settings)	6/14

Snyder et al.1995 (USA)	Schizophrenia or schizoaffective disorder	15 care home operators 30 patients	Case-control study (residential care homes)	8/14

**Table 18:** Characteristics and quality of studies included in autonomy and service user involvement domain

**Study (Country)**	**Type(s) of Mental Illness**	**Number of Participants**	**Type of Study** **(Type of Setting)**	**Quality Assessment**
Ahuja & Williams 2005 (UK)	Not specified	Not specified	Descriptive review (setting not specified)	4/14

Lewis 1995 (USA)	Severe mental illness	Not applicable	Descriptive reivew (nursing home)	5/14

Linhorst & Eckert 2002 (USA)	Mostly schizophrenia	Not specified	Descriptive review (psychiatric hospital)	4/14

Linhorst et al. 2005 (USA)	Severe mental illness	Not applicable	Qualitative study (psychiatric hospital)	2/5

Simpson & House 2002 (UK)	Not specified	3796	Systematic review (13 studies) (setting not specified)	13/14

Timko et al. 1993 (USA)	Schizophrenia or organic brain syndrome	403	RCT (psychiatric hospital and nursing home)	12/14

**Table 19:** Characteristics and quality of studies included in staff training and support domain

**Study (Country)**	**Type(s) of Mental Illness**	**Number of Participants**	**Type of Study** **(Type of Setting)**	**Quality Assessment**
Alexander et al. 2005 (USA)	Severe mental illness	1638	Cross-sectional study (inpatient settings)	9/14

Bradshaw et al. 2007 (UK)	Not specified	23 mental health nurses	Before and after study (setting not specified)	8/14

Corrigan et al. 2001 (USA)	Severe mental illness	Not specified	Descriptive review (setting not specified)	6/14

Linhorst 1995 (USA)	Severe and persistent mental illness	7 focus group members	Focus group study (long-term inpatient settings)	8/14; 3/5

Sowers 2005 (USA)	Not specified	Not applicable	Clinical guidance (setting not specified)	Not applicable

**Table 20:** Characteristics and quality of studies included in clinical governance domain

**Study (Country)**	**Type(s) of Mental Illness**	**Number of Participants**	**Type of Study** **(Type of Setting)**	**Quality Assessment**
Cape & Barkham 2002 (UK)	Not specified	Not specified	Systematic review (120 studies) (setting not specified)	5/14

Janssen et al. 2005 (Germany)	Schizophrenia	Not specified	Cohort study (psychiatric hospitals)	12/14

Most (n = 77) included papers used quantitative research methods. Of these, 24 were systematic reviews or meta-analyses and 19 were descriptive reviews. Three papers used qualitative methods and two used both qualitative and quantitative methods. Six papers were clinical guidelines. The types and number of studies relevant to each domain are shown in Table 21. Where studies used mixed methods they are counted only once in the table as quantitative studies.

**Table 21:** Study types and number of studies in each domain

**Domain**	**Systematic Reviews/Meta-analyses**		**RCTs**	**Qualitative Studies**	**Other Studies**	**Clinical Guidance**
	**Number of Reviews**	**Total Number of studies**				
**Living Conditions**	**2**	**58**	**0**	**0**	**15**	**1**

**Interventions for the Treatment of Schizophrenia**						

CBT	**2**	**39**	**2**	**0**	**0**	**2**

Family Interventions and Psychoeducation	**4**	**117**	**2**	**0**	**4**	**2**

Vocational Therapy	**3**	**46**	**0**	**0**	**3**	**2**

Social Skills Training	**3**	**33**	**0**	**0**	**2**	**2**

Cognitive Remediation	**2**	**24**	**1**	**0**	**0**	**0**

Arts Therapies	**3**	**11**	**0**	**0**	**0**	**0**

Integrated Therapy	**1**	**30**	**0**	**0**	**2**	**0**

Treatment of Comorbid Substance Misuse	**0**	**0**	**0**	**0**	**1**	**2**

Medication Management	**0**	**0**	**0**	**0**	**0**	**2**

Compliance Therapy	**1**	**1**	**4**	**0**	**1**	**0**

Occupational Therapy	**0**	**0**	**1**	**0**	**1**	**0**

Supportive Therapy	**1**	**21**	**0**	**0**	**0**	**0**

Coping Skills Training	**0**	**0**	**2**	**0**	**0**	**0**

**Physical Health**	**0**	**0**	**0**	**0**	**7**	**2**

**Restraint and Seclusion**	**4**	**275**	**0**	**1**	**9**	**4**

**Therapeutic Relationship**	**1**	**7**	**0**	**1**	**12**	**0**

**Service User Involvement and Autonomy**	**1**	**13**	**1**	**1**	**3**	**0**

**Staff Training and Support**	**0**	**0**	**0**	**0**	**4**	**1**

**Clinical Governance**	**1**	**Not specified**	**0**	**0**	**1**	**0**

### Quality assessment

Scores ranged from 2-5 for qualitative studies and 4-14 for quantitative studies. Scores for studies relevant to a particular domain can be found in Tables 1, 2, 3, 4, 5, 6, 7, 8, 9, 10, 11, 12, 13, 14, 15, 16, 17, 18, 19 and 20.

## Main Findings

The main findings from papers relevant to each domain are presented hierarchically, based on the quality of the papers, with findings from better quality papers presented first, followed by papers of weaker quality. Settings are reported as described in the papers.

### Living Conditions

Descriptions of the 18 studies relevant to living conditions can be found in Table 1.

#### Restrictiveness and setting

The American Psychological Association's (APA) guidelines for the treatment of schizophrenia suggest that, where patients require treatment in a residential facility, this should be in the least restrictive setting that will ensure patient safety and allow for effective treatment [22]. Overall, community residential facilities have been found to be less regimented than hospital wards and more facilitative of patient autonomy [23-25]. Hawthorne et al [26] examined two community residential facilities in America which emphasized provision of treatment in the least restrictive environment and positive staff-patient relationships. In a repeated measures design, where patients acted as their own control, patient functioning significantly increased and rehospitalisation significantly decreased in less restrictive settings even when patient morbidity was taken into account.

A number of studies have found that the majority of patients with longer term mental health problems prefer living in community, rather than hospital, settings [18,23,24,27,28]. Community settings have also been reported to be associated with better client outcomes than hospital settings [29]. In a national study of community-based residential facilities for people with mental health problems in Italy, facilities with higher levels of restrictiveness and fewer links with community-based activities experienced higher rates of hospital readmission [30]. A Danish study found that community residential facilities were better able to promote residents' activities both within the facility and in the community than hospital-based psychiatric rehabilitation units [31]. Residents of a community hostel, which emphasised individualised care, were found to have a better quality of life and greater freedom compared to patients in hospital-based rehabilitation units with similar levels of psychopathology and impairment [23]. The hostel also had the highest rating of rehabilitation environment quality, with lower social distance between staff and residents, greater flexibility and greater promotion of community integration for its residents.

In a descriptive review of community residential programmes, patient characteristics were reported to have a weaker correlation with positive outcomes than environmental factors [29]. In "board and care" homes in the USA, a positive social climate characterised as cohesive, organised, comfortable and encouraging of residents' independence and involvement in decision making, was found to be associated with greater resident satisfaction with their living situation [32]. High levels of resident involvement, support, spontaneity, autonomy, organisation and programme clarity have been cited as important components of environmental quality in group homes by both staff and residents [33]. Similar elements have been found to be important for greater therapeutic alliance between staff and patients in inpatient settings [34]. Brunt and Hansson [33] also found that security, physical (built) environment and social interaction were considered important by both residents and staff but staff more often stressed the importance of supporting residents to gain practical skills. However, Cournos [29] found that concordance between staff and residents about the importance of specific environmental characteristics was only weakly correlated with resident outcomes.

#### Cultural context

In a study comparing community-based residential facilities for people with mental health problems in Andalusia (Spain) and London (UK), Spanish facilities were found to be more restrictive with more rules and less privacy [18]. However, Spanish residents had more favourable views than their English counterparts on their individual progress and enjoyment of the company of other residents, greater acceptance of house rules and routine and they reported greater benefits from their activities and medication. Spanish residences were closer to community amenities but twice as many UK participants reported involvement in community activities (such as attending day centres or sheltered employment) whereas Spanish residents made greater use of indoor recreational activities.

#### Number of residents

There is no clear evidence on the optimal number of residents in community-based residential mental health facilities. A study carried out in the USA found no association between the number of residents per facility and residents' integration in activities within the facility, after adjustment for other factors [25]. Another study found the number of residents in community-based "board and care" homes for veterans in the USA was positively correlated with social functioning in the community [32]. Although, an optimal number will depend upon "the prevalent philosophy of care, available resources and population need", density of occupation (the ratio of residents to available space) rather than a recommended specific number of residents is considered a better guide, since increased density increases residents' stress and decreases their privacy and control over their environment [35].

#### Physical environment

The effect of the physical environment on patient outcomes was examined in a systematic review of 30 controlled trials [36]. Participants included those with mental health as well as physical health problems. No trials were identified that exclusively investigated wall colour, pictures, plants, gardens, floor coverings or room size. Eleven trials investigated the effect of renovation or redecoration of a whole ward or treatment area on participants' social functioning and symptoms. Inconsistent findings were reported. Two trials found that the amount and timing of access to sunlight was associated with a reduced length of admission for depressed patients. One trial carried out in a psychiatric unit showed that seating arrangements in common areas that encouraged interaction (e.g. seating around small tables) increased patients' social interaction.

Baker and Douglas [37] carried out a large study in New York to investigate outcomes for people with mental health problems living in supported and unsupported community housing of varying condition (assessed by observer ratings of the property's location, exterior condition, interior condition and the condition of their personal property). Those in supported housing where physical conditions were rated below average displayed a significant increase in maladaptive behaviour over the nine-month study period compared to those in housing of average or above average quality.

In a systematic review of 28 studies of supported housing for people with mental health problems, both the quality of the physical environment and the degree of privacy were found to mediate patient outcomes [27]. Corrigan [38] investigated mental health inpatients' satisfaction with their accommodation and found lack of privacy to be a major concern, specifically having a place to be alone and secure storage for personal items.

### Interventions for the Treatment of Schizophrenia

The APA recommends that realistic treatment outcomes for individuals with a diagnosis of schizophrenia are identified and assessed using standardised outcome measures [22]. Although there is good evidence for the efficacy of a number of interventions, those selected should be tailored to the patient/resident's individual needs.

#### Cognitive behavioural therapy

Meta-analyses of cognitive behavioural therapy (CBT) for patients with schizophrenia and other related psychoses have found consistent evidence for its efficacy [39,40]. Descriptions of these studies as well as other evaluations of CBT can be found in Table 2. Pilling et al [39] found CBT had a small effect on improving positive symptoms during treatment (n = 273, effect size 0.27, CI 0.15, 0.49; NNT 5, CI 4, 9) and nine to 18 months after treatment (n = 119, effect size 0.25, CI 0.10, 0.64; NNT 6, CI 3, 27) but was not associated with reduced relapse rates. Pfammatter et al [40] examined three meta-analyses of CBT's effect on positive symptoms. The effect varied from small (effect size 0.33, CI 0.14, 0.51) to large (effect size 0.93, CI not reported). In their own meta-analysis of 17 randomised controlled trials (RCTs) they reported that consistent (but small) effects for CBT could only be established when provided to individuals with persistent positive symptoms (during treatment: n = 486, effect size 0.47, CI 0.29, 0.65; post-treatment: n = 335, effect size 0.39, CI 0.17, 0.61) [40].

Turkington et al [41] carried out a multicentre RCT to investigate the efficacy of CBT for patients with schizophrenia who had ongoing positive and/or negative symptoms or were at risk of relapse and found CBT to be associated with improved insight (ANCOVA 0.711, CI 0.11, 1.31, *p *= 0.021) and fewer negative symptoms (eta^2 ^-0.773, CI -1.27, -0.28, *p *= 0.002) than participants assigned to usual care at 12-month follow-up. It was also found to be protective against depression and relapse.

An RCT of group CBT for people with schizophrenia found no effect in terms of improvement in symptoms, functioning or relapse rates, but a significant increase in self esteem (n = 94, effect size -1.51, CI -2.84, -0.18) and decrease in hopelessness (n = 94, effect size -1.62, CI -3.06, -0.18) at 12-month follow-up [42].

Both NICE [43] and the APA [22] recommend offering CBT to individuals with schizophrenia, especially those with persistent positive symptoms, with NICE recommending treatment over at least six months comprising at least ten sessions.

#### Family Interventions and Psychoeducation

Many family interventions involve psychoeducation and many trials of psychoeducation involve family members. Therefore, we have included studies of both family interventions and psychoeducation in this section (see Table 3).

Meta-analyses show that, compared to usual care, family interventions (including psychoeducation, crisis management work and problem solving) for people with a diagnosis of schizophrenia reduce the risk of relapse (n = 3838, effect size 0.42, CI 0.35, 0.49) [40] and readmission (6-12 month follow-up: n = 3789, effect size 0.22, CI 0.14, 0.29; 18-24 month follow-up: n = 445, effect size 0.51, CI 0.32, 0.70) [40] and improve medication adherence (n = 393, effect size 0.63, CI 0.40, 1.01; NNT 10, CI 6, 90) [39] (n = 369, RR 0.74 CI 0.6, 0.9; NNT 7 CI 4, 19) [44].

Family interventions that include patients and their relatives are more effective than those for relatives alone [43]. Both single and multiple family interventions are efficacious but drop-out from multiple family interventions is high. Lehman et al [22] suggest the best time to engage families is during the acute phase of the illness or at times of crisis.

In a meta-analysis of 31 RCTs of family psychoeducation carried out by Pfammatter and colleagues [40], improvements were shown in family members' understanding of the disorder (n = 3662, effect size 0.39, CI 0.31, 0.46) and expressed emotion (n = 284, effect size 0.59, CI 0.36, 0.83) and in patient's social functioning (n = 3362, effect size 0.38, CI 0.30, 0.46) and general psychopathology (n = 178, effect size 0.40, CI 0.10, 0.70).

In a Cochrane review of 10 RCTs of psychoeducation for service users, which included interventions where family members also participated, psychoeducation was found to significantly decrease relapse rates at nine to 18 months follow-up (n = 720, RR 10.8, CI 0.70, 0.92; NNT 9, CI 6, 22) and increase global psychosocial functioning (Weighted Mean Difference (WMD) 5.2, CI -8.8, -1.7) at one year follow-up [45].

An RCT examining the effectiveness of psychoeducation provided to patients and families in separate groups compared to standard care found patients in the experimental group had significantly lower rehospitalisation rates than the standard care group at 12 (N = 163, RR 0.56, CI .033, 0.92) and 24 (N = 153, RR 0.70, CI 0.50, 0.97) month follow-up [46].

Carrà et al [47] found no statistically significant differences in patients' relapse or readmission rates in an RCT in which families were assigned to attend either a psychoeducation group with the patient, a psychoeducation group with the patient plus a support group without the patient, or treatment as usual. Patient adherence with standard care was better for families who received the psychoeducation plus support group intervention, although carer burden increased. However, several studies have found that both service users and their family members receiving psychoeducation show an improved level of knowledge about the relevant psychiatric condition [45,48] but no consistent improvement in insight or adherence to medication [45].

As well as reduced relapse rates and improved symptoms and social functioning, other reported benefits of multiple family psychoeducation groups are improved well-being for family members and increased service user participation in vocational rehabilitation and competitive employment [49].

Although psychoeducation has been shown to have beneficial effects on patient outcome, it is not regularly provided in inpatient care [48]. Rummel-Kluge et al [21] used a large postal survey to investigate difficulties in implementing a psychoeducational intervention in psychiatric hospitals in Germany, Austria and Switzerland. Although 86% of the institutions offered psychoeducation, only 21% of patients with schizophrenia and 2% of their family members had received the intervention in the previous year. Staff stated they lacked resources and training.

Clinical guidance from the UK [43] and US [50] recommends family interventions last over six months and with a minimum of ten sessions [43,50].

#### Vocational therapy

Supported employment is an approach to improve vocational functioning among people with various mental health problems including schizophrenia [22]. Evidence is strongest for programs that encourage direct entry into competitive employment and provide individualised workplace support rather than models which offer step-wise progression towards employment [51,52]. In two meta-analyses of different approaches to vocational rehabilitation, supported employment was found to be three to four times more successful in achieving competitive employment than other forms of vocational training such as sheltered workshops, psychosocial rehabilitation work programmes and transitional employment schemes (RR 0.76, CI 0.64, 0.89; NNT 4.5, CI 4.48, 4.63) [53] (OR 4.14, CI 1.73, 9.93) [54]. Descriptions of studies relevant to vocational therapy can be found in Table 4.

Individual Placement and Support (IPS), a specific, manualised version of supported employment, has been shown to be more effective than prevocational training in terms of participants achieving competitive employment (n = 295, RR 0.79, CI 0.70, 0.89; NNT 5.5, CI 3.6, 10.2) [53] and their number of days in employment but there is insufficient evidence as to whether IPS is more effective than other less carefully specified forms of supported employment [53,54]. The components of IPS that are most beneficial are: rapid job search; elimination of prevocational preparation; sensitivity to the client's job preferences, strengths and work experience; integration with mental health services and time-unlimited support [50,52].

Integration of supported employment programmes within other mental health services is more successful in engaging and retaining clients in vocational rehabilitation than when these services are separately provided. Integrated programmes also reduce problems with communication between services and raise mental health staff's awareness of the achievability of clients' vocational goals [55].

Supported employment is recommended by the APA [22]. In the UK, NICE [43] recommends the provision of supported employment for individuals who wish to work. However, they also recommend that other vocational rehabilitation resources are available for those who are unable to work.

#### Social skills training

Social skills training (SST) aims to improve social functioning for people with a diagnosis of schizophrenia by teaching them skills to improve their social performance in activities of daily living, employment, relationships and leisure [56]. Descriptions of studies examining SST can be found in Table 5.

The effectiveness of SST has been examined in two meta-analyses [40,57] which reached different conclusions regarding evidence for its efficacy. Pilling et al. [57] included nine RCTs of SST and found no clear evidence of benefit for relapse rates, global adjustment, social functioning, quality of life or treatment adherence. In contrast, Pfammatter and colleagues' [40] meta-analysis of 19 SST studies (quasi-experimental studies as well as RCTs) found beneficial effects for the acquisition of social skills (during treatment: n = 688, effect size 0.77, CI 0.62, 0.93; post-treatment: n = 295, effect size 0.52, CI 0.28, 0.77), improvement in social functioning (during treatment: n = 342, effect size 0.39, CI 0.19, 0.59; post-treatment: n = 210, effect size 0.32, CI 0.08, 0.56) and reduced hospitalisation (post-treatment: n = 110, effect size 0.48, CI 0.11, 0.86).

Bustillo et al [56] included five SST studies (two were RCTs) in a systematic review of psychosocial treatment for schizophrenia. They noted that although social skills were usually enhanced when assessed, this did not generalise to social competence in the community.

Roder et al [19,20] carried out an evaluation of a four stage skills training program focused on improving either recreational skills, vocational skills or residential skills for patients at eight institutions in Germany, Switzerland and Austria. Participants were assigned to the group that most addressed their goal of interest and matched for age, psychopathology, duration of illness and motivation. Group and individual sessions, in-vivo exercises and homework assignments were used to focus on clients' most frequent problems. Small to medium effect sizes for cognitive and social functioning for all three programs were found at three (recreational skills: effect size 0.35, CI not reported, vocational skills: effect size 0.40, CI not reported, residential skills: 0.51, CI not reported), six (recreational skills: effect size 0.48, CI not reported, vocational skills: effect size 0.47, CI not reported, residential skills: effect size 0.60, CI not reported) and 12-month (recreational skills: effect size 0.58, CI not reported, vocational skills: effect size 0.66, CI not reported, residential skills: effect size 0.73, CI not reported) follow-up.

The American Psychiatric Association states "SST may be helpful in addressing functional impairments with social skills of activities of daily living" [22] but it is not recommended by NICE [43].

#### Cognitive remediation

A meta-analysis of five RCTs of cognitive remediation plus standard care found no benefit in terms of attention, verbal memory, visual memory, mental state or executive functioning over standard care alone [57]. However, in Pfammatter et al's [40] review of six meta-analyses small to medium effects of cognitive remediation on general cognitive functioning were found, as well as an indication of a possible transfer of these effects to social functioning. Through a further meta-analysis of 19 studies, cognitive remediation was found to have a small effect on attention (n = 539, effect size 0.32, CI 0.15, 0.49), executive functioning (n = 606, effect size 0.28, CI 0.12, 0.44), memory (n = 704, effect size 0.36, CI 0.20, 0.51) and social cognition (n = 228, effect size 0.40, CI 0.13, 0.68) [40]. A moderate transfer effect on social functioning (n = 306, effect size 0.49, CI 0.27, 0.70) and small reductions in overall psychopathology (n = 452, effect size 0.20, CI 0.01, 0.38) and negative symptoms (n = 394, effect size 0.24, CI 0.04, 0.44) were found. Descriptions of studies relevant to cognitive remediation can be found in Table 6.

More recently, Wykes et al [58] conducted a single blind RCT comparing outcomes for participants assigned to receive 40 sessions of cognitive remediation therapy with participants receiving standard care. A small effect on working memory was found (effect size 0.34, CI 0.1, 0.55) but there were no differences between groups in social functioning.

#### Arts therapies

Gold et al [59] conducted a meta-analysis of four RCTs comparing music therapy for inpatients with a diagnosis of schizophrenia plus standard care with standard care alone. A minimum of 20 sessions was associated with significant improvement in positive and negative symptoms while findings for interventions with less than 20 sessions were inconclusive. Recipients of music therapy had significantly improved global functioning (n = 72, RR 0.10, CI 0.03, 0.31; NNT 2, CI 1, 2) and individuals receiving "high dose" music therapy (average 78 sessions) showed significant improvement in social functioning (Standardised Mean Difference -0.78, CI -1.27, -0.28). Descriptions of studies relevant to arts therapies can be found in Table 7.

A Cochrane review and meta-analysis of two RCTs of art therapy for people with schizophrenia found marginally beneficial effects on mental state but no effect on social functioning or quality of life [60]. The need for further RCTs was recommended. A Cochrane review of drama therapy identified five RCTs but, with minimal extractable data, no conclusions regarding efficacy could be made [61].

#### Integrated therapy

Integrated therapy, which incorporates psychosocial and pharmacological interventions, has been evaluated in a number of studies. Descriptions of these studies can be found in Table 8.

The most widely implemented model is integrated psychological therapy (IPT), a group-based CBT programme for people with schizophrenia, which integrates neurocognitive remediation with social cognition, problem solving and social skills training. Roder et al [62] conducted a meta-analysis of 30 studies of IPT, then a second meta-analysis using only the highest quality studies (n = 7) to determine whether or not the results would confirm the findings of the first meta-analysis. In comparison to standard care or placebo-attention control interventions, medium effect sizes were reported for participants who received IPT for global effect (N = 253, effect size 0.65, CI 0.39, 0.74) and psychopathology (N = 638, effect size 0.58, CI 0.39, 0.61), small to medium effect sizes were reported for functioning (neurocognition: N = 633, effect size 0.61, CI 0.43, 0.65; psychosocial functioning: N = 530, effect size 0.43, CI 0.29, 0.54) and small effect sizes for symptoms (positive symptoms: N = 424, effect size 0.42, CI 0.32, 0.60; negative symptoms: N = 277 effect size 0.46, CI 0.24, 0.57). Inpatients showed greater improvement at follow-up than outpatients (inpatient weighted effect size [at 10-month follow-up] 0.79, CI 0.43, 1.16 vs. outpatient weighted effect size [at 7.5-month follow-up] 0.44, CI 0.07, 0.80). Studies including only social skills training and problem solving sub-programmes showed no effect on neurocognition. Effects at follow-up were stronger when all five sub-programmes (cognitive differentiation, social perception, verbal communication, social skills and interpersonal problem solving) were provided. Longer term therapy had a beneficial effect on functional outcome. However, individuals with longer illness durations were less likely to benefit from IPT.

Treatment programmes that specifically combine pharmacological and psychosocial interventions (including supportive psychotherapy, family interventions, SST and CBT) have been reported to have additive positive outcomes for patient with schizophrenia [63].

The Illness Management and Recovery programme, a medication adherence intervention comprised of SST, coping skills training, recovery strategies, a relapse prevention plan and a cognitive behavioural approach, was developed by Gingerich and Mueser [64]. The programme was found to be beneficial in individuals receiving psychiatric care in non-residential community settings who reported high levels of satisfaction and found the programme to be useful, respectful, helpful for managing their symptoms and beneficial in making progress towards their goals [65].

#### Treatment of comorbid substance misuse

In their review of treatments for people with severe mental illnesses and co-occurring substance misuse, Drake et al [66] concluded that the available evidence supported the integration of mental health and substance misuse interventions into a single care package. Patient-centred programmes were especially effective as they addressed the individual's stage of motivation for change. Programmes that incorporate some form of motivational counselling and outreach to engage the individual were recommended [66].

An integrated approach is supported by the APA [22]. Consensus expert guidance also endorses integrated treatment plus screening all patients with psychiatric symptoms for substance misuse and assessing the type, amount and mode of substance use; the client's motivation for change; physical sequelae of substance use, medication and psychosocial treatments including motivational therapy, modified CBT and relapse prevention [67]. Descriptions of studies relevant to the treatment of comorbid substance misuse can be found in Table 9.

#### Medication management

Evidence-based clinical guidance and treatment algorithms assist clinicians in the choice and administration of antipsychotic medications [22,43]. These are described in Table 10. These guidelines also stress the importance of discussion and negotiation with the patient about the choice of medication, changes in medication and regular review of its effects and side effects [22,43].

#### Compliance therapy

A Cochrane review of compliance therapy for patients with schizophrenia [68] included only one RCT comparing it with non-specific counselling [69]. The manualised intervention was based on cognitive behavioural interventions and included aspects of motivational therapy, cognitive therapy and psychoeducation. No evidence of efficacy was found. Descriptions of studies relevant to compliance therapy can be found in Table 11.

Compliance therapy comprising four to six individual sessions exploring the pros and cons of patients' medications is more effective than supportive counselling [70], with advantages in terms of increased compliance (mean difference 19%; CI 0.9, 1.6), insight (mean difference 18.8%; CI 12.3, 25.2), attitudes towards medication (mean difference 15.6%; CI 2.5, 7.2) and global functioning (mean difference 2.4%) from baseline to 18 months after the end of the intervention.

A clinician administered, behaviourally-oriented programme on medication management and patient outcomes lead to improvements in patients' knowledge about medication, medication management and compliance [71]. Eckman et al [72] subsequently compared outcomes for male veterans receiving social and independent living skills training in groups or supportive group psychotherapy in an RCT. In group by trial analyses, individuals assigned to skills training showed significant improvement in medication management (F(1, 34) = 75.1, *p *< 0.0001) and symptom management (F(1, 32) = 36.23, *p *< 0.0001). Significant differences in medication management in favour of skills training were found between groups at six-month follow-up (F(1, 30) = 56.45, *p *< 0.0001) and 12 (F(1, 25) = 40.28, *p *< 0.0001). Significant differences between groups in favour of skills training were also found for symptom management at six-months follow-up (F(1, 28) = 6.34, *p *< 0.02) and 12 (F(1, 22) = 9.41, *p *< 0.005).

Whether psychoeducation on medication is structured or not seems to make no difference to patient knowledge about medication or positive attitudes [73].

An RCT comparing medication adherence for patients receiving either psychoeducation (medication information talks given by medical or nursing staff) or treatment as usual found significantly more control group participants were non-adherent at five-month follow-up based on pill count (N = 32, effect size 0.13, CI 0.03, 0.53, *p *< 0.001) and urine testing (N = 38, effect size, 0.22, CI 0.03, 1.73, p < 0.001) [74].

Streicker et al [75] investigated effects of a ten session medication education programme on participants assessed as non-adherent at baseline in a non-randomised controlled study and found that those who received the intervention gained knowledge about their medication but were more likely to be non-adherent post-intervention and at follow-up and as likely to be admitted to hospital as controls who received no intervention.

#### Occupational therapy

There is very little evidence on the effectiveness of occupational therapy in this patient group. Descriptions of included studies can be found in Table 12.

In a small (N = 26), non-blinded RCT carried out in Brazil, participants were assigned to six months of occupational therapy plus clozapine or clozapine alone [76]. Outcomes included activity, symptoms, social interaction and personal care. In an intention-to-treat analysis at six months, a combined rating of symptoms and social functioning showed significant advantage for those who received occupational therapy (effect size -1.44, *p *= 0.001).

Oka et al [77] retrospectively examined "before and after" outcomes over 17 years, for 52 patients with a diagnosis of schizophrenia discharged from a Japanese hospital between 1976 and 1990 who had taken part in inpatient occupational therapy, six days per week, in an integrated supported employment programme prior to discharge. Measures of social adjustment and rehospitalisation showed significant improvement pre and post-treatment.

#### Supportive therapy

In a Cochrane review, Buckley and colleagues [78] examined the effectiveness of supportive therapy for schizophrenia, defined as any "one-to-one" intervention which aimed to improve or maintain the patient's functioning (see Table 13 for study descriptions). There was no evidence that supportive therapy was more beneficial than standard care or other psychological or psychosocial therapies and, in fact, supportive therapy was more likely to cause social impairment (n = 39, RR 1.46, CI 1.0, 2.0; Number Needed to Harm (NNH) 4, CI 3, 39), treatment-related early termination (n = 151, RR 2.15, CI 1.1, 4.3; NNH 8, CI 3, 128), an episode of affective symptoms (n = 151, RR 1.84, CI 1.6, 2.9; NNT 5, CI 3, 27) and poor medication compliance between 13 to 26 weeks follow-up (n = 39, RR 2.63, CI 1.3, 5.4; NNT 3, CI 2, 12).

Significant differences favouring other psychological or psychosocial therapies were found: reduced chance of relapse (n = 39, RR 1.87, CI 1.1, 3.2; NNT 3, CI 3, 21) and hospitalisation (n = 241, RR 2.12, CI 1.2, 3.6; NNT 8, CI 4, 35) at six-month follow-up; greater general functioning at three months (n = 70, WMD -9.5, CI -16.1, -2.9) and six months (n = 67, WMD -12.6, CI -19.4, -5.8) follow-up; better general mental state (n = 194, RR 1.27, CI 1.0, 1.5; NNT 7, CI 4, 43), reduced symptoms (n = 12, WMD 17.10, CI 13.8, 20.4), better attitudes to medication (n = 74, WMD -4.50, CI -6.8, -2.2) and greater treatment satisfaction (n = 45, RR 3.19, CI 1.0, 10.1; NNT 4, CI 2, 736) were found at six-month follow-up [78].

#### Coping skills training

The effectiveness of a coping skills training programme for service users comprising 24 twice weekly sessions (n = 55) was tested against treatment as usual (n = 44) in an RCT [79]. The approach consisted of training in seven components for coping with daily life: identification of symptoms; cognitive appraisal of stress, change and resources; selection of a coping strategy; use of strategy and evaluation of the results. Although no difference between groups was found, participants in the experimental group showed greater change scores over time as compared to the control group in hygiene (F(2,120) = 4.25, *p *< 0.05), self-esteem (F(2,140) = 3.08, *p *< 0.05) and delusions (F(2,132) = 3.16, *p *< 0.05). However, these findings are limited as the analyses were performed secondary to the main trial. Descriptions of relevant studies can be found in Table 14.

The effectiveness of a training programme focused on encouraging service users' self-esteem and empowerment was assessed in an RCT [80]. Although the intervention had no significant advantage compared to the control intervention, group by time analyses found a significant improvement in coping (F(1,70) = 4.01, *p *< 0.05). Positive symptoms were also significantly decreased in group by time analyses in the experimental arm (F(1,68) = 7.72, *p *< 0.01) but at six-month follow-up had reverted to baseline scores. The intervention was most beneficial in improving coping skills among participants with severe symptoms and lower functioning.

### Physical Health

Screening and treatment for physical health problems is an important aspect of care for individuals with severe mental health problems receiving longer term care as the client group tends to have less healthy lifestyles than the general population (poor diet, lack of physical exercise, smoking and substance misuse). Those with a diagnosis of schizophrenia are at greater risk for negative health outcomes than individuals with other mental health diagnoses [81,82]. Patients with serious mental health problems are also at increased risk of mortality from respiratory disease, cardiovascular disease and cancer [81,82]. As well as unhealthy lifestyle choices, some of their excess cardiovascular risk may be attributable to obesity, and lipid and glucose dysregulation related to their antipsychotic medication [83]. Descriptions of studies relevant to physical health can be found in Table 15.

Individuals with schizophrenia or similar mental health problems have been found to be willing to attend for cardiovascular risk assessment in primary care settings [84]. However, screening for physical health problems amongst inpatients with serious mental health problems is poor, with only one quarter of diseases being detected and a high proportion of mental symptoms being wrongly attributed to functional mental illness rather than organic causes [85].

In a survey of medical directors from 102 public psychiatric hospitals in the USA, over one-third of respondents believed their patients usually received poor medical care when transferred to acute medical services [86]. Patients with serious mental illnesses residing in longer term inpatient rehabilitation wards have been found to have poorer dental health than the general population [87].

To prevent and detect physical health problems among people with schizophrenia, health promotion and regular physical assessment have been recommended by advisory bodies in the UK and USA based on extensive literature reviews [22,43]. This should include advice about diet, exercise and smoking cessation as well as regular monitoring of weight and screening for extrapyramidal side effects, diabetes, hyperlipidaemia, hyperprolactinaemia and cardiac problems (using ECGs) for those prescribed antipsychotic medications. Ideally, physical assessments should be conducted at admission to a mental health unit or as soon as the patient is able to consent [85]. Primary and secondary health care services should agree which service will be responsible for the assessment and monitoring of physical health. Secondary mental health services should take on this role if the individual is not in receipt of primary health care.

### Restraint and Seclusion

The proportion of patients who have been secluded or restrained in psychiatric settings has been reported to be between two and 51 per cent [88]. Factors associated with different rates include the definition used, the type of facility (acute, hospital-based settings have higher rates than longer term and community-based settings), the philosophy and culture of the institution, the physical layout of the building and the level of staffing [88]. Wards with fewer male staff have been found to have higher seclusion rates but this is not a consistent finding [88]. In a Norwegian hospital, Wynn [89] found that the use of different restraint and seclusion methods varied depending on patient characteristics such as age, gender and diagnosis. Physical restraint was used more often with young, male, non-psychotic populations. Older males with organic psychotic disorders were most likely to be secluded [89]. However, Palazzolo et al [90] carried out a non-systematic review of the literature on restraint and seclusion and found no consistent staff or client characteristics associated with its use. Descriptions of studies relevant to restraint and seclusion can be found in Table 16.

Two reviews have concluded that there is insufficient evidence to determine the efficacy of restraint and seclusion [88,91]. A recent Cochrane review [92] identified no RCTs investigating the efficacy of restraint and seclusion for people with mental health problems which met their inclusion criteria. A complex intervention to reduce the use of restraint and seclusion in a New York hospital succeeded, but the number of violent assaults by patients on staff and other patients increased [93].

Despite the lack of conclusive evidence for the efficacy of seclusion or restraint and the finding that its use is traumatic for staff, it has been suggested that it may be impossible to provide a programme of treatment for individuals with severe mental health problems without it [88,90,91,94]. Patients have also reported negative experiences of the use of restraint and seclusion [88,94,95] with many feeling the interventions were overused, not always justified [91,95] and harmful to the therapeutic alliance [94,95]. However, some indicated that the use of restraint was reasonable and its effect was calming [95]. It is generally agreed that restraint and seclusion interventions should be avoided unless absolutely necessary [88].

Staff training in protocols that define situations that warrant the use of seclusion or restraint and provide a clear algorithm are recommended in studies as well as clinical guidance from Canada, New Zealand and Australia, the USA and the UK [17,22,43,91,96]. De-escalation techniques should be implemented early on [22,43,96]. The interventions should be supervised and the patient monitored throughout [43]. Debriefing for staff and patients should be provided following the use of restraint or seclusion and the event should be documented in the client's records [43,91,95]. Training in prediction and prevention of violence may also help reduce the need for seclusion and restraint [90,93,97,98].

Gaskin et al [97] carried out a literature review of 16 non-randomised studies of interventions to reduce the use of seclusion in psychiatric settings. Typically, multiple interventions were used including legislation, increased staffing, emergency response teams, staff education, staff training, supervision, pharmacological interventions, changes to the therapeutic environment to increase collaboration between patients and staff, involvement of patients more actively in seclusion reduction interventions and rotation of staff to work less intensively with acutely unwell patients. In studies where strategies to engage and empower patients were employed, there was less need for the use of restraint and seclusion. In addition, setting expectations regarding use, reviewing local policies pertaining to restraint and seclusion and providing staff with additional professional resources had beneficial effects on decreasing its use.

A study carried out in Poland examined the use of physical restraint in eleven locked wards between 1989 and 1996 in order to investigate any change in practice secondary to the implementation of a new Mental Health Act in 1995 and associated national guidance on the use of restraint [99]. There were more episodes of restraint in 1996 but the number of episodes per patient had reduced and more were in response to physical aggression than in 1989. A retrospective study carried out in four hospitals in the Netherlands found that nurses with greater training and experience were less likely to use seclusion than their less well trained colleagues [100].

Crisis or rapid response teams that meet within a defined time period of a restraint or seclusion (usually 24 hours) to review the appropriateness of its use and suggest alternative approaches have been shown to reduce the use of restraint and seclusion [93,98,101,102].

### Therapeutic Relationship

The therapeutic relationship between service users and staff is one of the most potent predictors of patient outcomes in psychotherapy [103]. Positive therapeutic relationships between patients and staff have also been shown to be associated with improved outcomes for those with severe mental illnesses [104-106] with several studies finding a significant relationship between patient functioning and therapeutic alliance [34,107,108]. Descriptions of studies relevant to the therapeutic relationship can be found in Table 17.

McCabe and colleagues [109] investigated the relationship between patients' quality of life and their therapeutic relationship with staff and found a positive association for patients with longer term mental health problems compared to those undergoing their first episode of illness. The authors suggested that the therapeutic relationship may become increasingly important over time for this client group.

A case-control study investigating expressed emotion in residential care homes for people with mental health problems in Los Angeles, found high rates of critical comments from staff towards patients were negatively correlated with patient satisfaction and associated with increased levels of positive symptoms at 12-month follow-up [110]. However, greater critical comments were made towards patients whom staff felt could recover, whilst lower expressed emotion was directed towards the patients staff felt were unlikely to recover. Conversely, McCabe et al [104] found that mentally ill patients with less severe symptoms had more positive relationships with staff.

The quality of the therapeutic relationship is influenced by patient, staff and organisational factors. The professional background of staff may influence their therapeutic relationships due to their training, the theories underpinning their profession and the types of interventions they provide [103]. Therapists' perceptions of their therapeutic relationships with patients with schizophrenia were found to be more strongly correlated with patient outcome than patients' perceptions of the relationship [105]. In a qualitative study examining staff perceptions of factors related to therapeutic relationships, whether or not a patient was liked or disliked by staff was considered the most important factor in the amount of time the staff spent with that patient [111]. The likeability of staff is also important to the therapeutic alliance and has been shown to be positively associated with better patient functioning [108]. Organisational issues, such as excessive administration tasks, the occupational density of the unit, under staffing, workload and conflicts between staff also reduce staff contact with patients [111].

A lack of information and involvement in treatment is associated with greater patient and family dissatisfaction with services [112]. Client-centred care, where staff focus on the things that are important to the patient rather than the things considered important by the clinical team, is increasingly encouraged [103]. In a cross-sectional study comparing the goals of long-stay supported housing residents (N = 41) with those of staff, little or no agreement was found [28]. Client-centred care may therefore be particularly beneficial in improving therapeutic alliance when the goals of patients and staff diverge [28]. By engaging and involving patients in their own care, positive therapeutic relationships develop [103] and can improve patient outcomes [34,104-108].

The effectiveness of the implementation of the "Tidal Model" [113] in an inpatient psychiatric unit was investigated through a cross-sectional survey. The model involves a collaborative, client-centred therapeutic approach to enhance skills for recovery through regular goal setting and individualised care planning and review and its implementation was associated with improved staff and service user satisfaction and greater service user involvement in their care [114].

### Autonomy and Service User Involvement

The effect of involving service users in the delivery and evaluation of mental health services was assessed in a systematic review of five RCTs and seven comparative studies [115]. Of the twelve included studies, eight involved evaluations of service users as service providers, two considered service users involved as trainers of service providers and two considered involving service users as interviewers for the evaluation of services. No negative effects were found for service users employed as case managers in terms of their own symptoms, quality of life or functioning. Involvement improved clients' quality of life and social functioning. Involving service users as trainers resulted in trainees having a more positive attitude towards people with mental health problems. However, respondents reported lower levels of satisfaction when interviewed by other service users than when interviewed by non service users [115]. For descriptions of studies relevant to autonomy and service user involvement, see Table 18.

Linhorst et al [116] carried out a qualitative study involving clients and staff at a longer term psychiatric hospital in the United States to determine useful ways of including clients in organisational decision making. Three means of engaging clients were felt to be particularly useful: a consumer council; involving clients in formal policy reviews; including clients in the hospital's performance improvement system. Linhorst and Eckert's [117] descriptive review of service user involvement in service evaluation processes reports that this approach facilitates better understanding of service users' views and expectations, and increases their personal investment and involvement in service improvement.

In a descriptive review examining the involvement of service users and carers in staff training and service development, it was concluded that this approach could improve patient and service outcomes [118]. However, the authors point out the possibility of unintended consequences. They suggest professional educators might feel their authority was threatened by expert patients and patient educators might be perceived as promoting their own personal agenda rather than the intended training goals [118]. Training might also cause anxiety among service users and carers and it was therefore recommended that these issues be considered when planning training courses.

In a large cross-sectional study of residents of different types of specialist mental health residential care settings in the USA, a positive correlation between the degree of control given to residents over their daily lives and their satisfaction with life was found [119].

Lewis [120] provides a description of the ways in which residents of a residential care home for people with longer term mental health problems are actively involved in the running of the home (such as having a residents' committee, a residents' welcoming committee for new residents, a food committee, a residents' newsletter and involvement in orientation programmes for new staff). He also advocates the importance of facilitating activities of citizenship such as voting and accessing advocacy services [120]. Lewis [120] also recommends facilities form a leadership group comprised of consumers, family members, service providers, and administrators to increase the influence of resident involvement in decision making. However, residents should be given clear information about the extent of decision-making power they have when participating via these structures.

### Staff Training and Support

Alexander et al [121] examined the effect of inpatient staff team characteristics on patient outcomes in 40 units in 16 hospitals across the USA and found patients' functioning in activities of daily living was positively associated with the degree of multidisciplinarity of the team. For study descriptions see Table 19.

A before and after study, assessed the impact of clinical supervision on implementation of psychosocial interventions by nursing students who attended a formal psychosocial intervention training course [122]. Supervision was provided every two weeks by qualified nurses trained in supervision. Outcomes were compared to those from the previous year's cohort who received standard nursing education. Both groups showed significant increase in case management knowledge post training, but only the intervention group showed significantly increased knowledge about psychological interventions and schizophrenia. Patients receiving treatment from experimental group students had significantly reduced symptoms compared to baseline.

Two qualitative studies comprising focus groups of hospital administrators, superintendents and managers have recommended training in effective psychosocial interventions and the philosophy of rehabilitation [123] and recovery orientated practice [124] for staff who work with clients with longer term mental health problems. A review of strategies for the implementation of evidence-based practices for this client group cited lack of training as a key barrier [125]. In-service training focused on skills important in implementing evidence-based practice has been shown to improve staff attitudes towards new practices and increase skills, but many staff do not participate in training and those that do may lose the skills they have learned unless these are reinforced through supervision or "booster" sessions [125].

An intervention to improve team leadership was found to improve team leaders' supervisory feedback and was related to an increase in service user satisfaction and quality of life [125].

### Clinical Governance

In a cohort study carried out in Germany, Janssen et al [126] found that low clinical guideline adherence by staff was associated with poorer clinical outcomes for patients. Benchmarking was found to be important in improving service quality and adherence to treatment guidelines. Descriptions of relevant studies can be found in Table 20.

Cape and Barkham [127] conducted a systematic review to evaluate practice improvement methods for health care services. They describe the model of practice improvement as involving three main stages which operate in a continuous feedback loop: process guidance (such as education and training, evidence-based clinical guidelines, and clinical supervision); process monitoring (through clinical audit, clinical supervision and quality improvement); and outcomes management (outcomes monitoring, quality improvement and benchmarking). These components of service improvement were shown to be effective in changing professional practice and improving health outcomes but staff had to commit to clinical audits and feedback. Practice improvement methods focussing on more than one intervention were more effective than those concerned with single interventions.

## Discussion

Care provision for individuals with severe mental health problems in longer term hospital or community based settings has historically been based on professional opinion rather than scientific evidence. We intended to undertake a wide ranging review of the international literature that would have broad clinical appeal. We identified eight domains of care that accord with findings from a qualitative study of service users, carers, professionals, policy makers and other citizens in five European countries [128]. These were living conditions; interventions for schizophrenia; physical health; restraint and seclusion; staff training and support; therapeutic relationship; autonomy and service user involvement; and clinical governance.

### Interpretation

Our results indicate that the ideal institution would be based in the community, operate a flexible regime, maintain a low density of residents and maximise residents' privacy. Since the majority of service users in these settings have a diagnosis of schizophrenia, specific interventions with high efficacy (CBT, family interventions involving psychoeducation and integrated supported employment) are key to positive outcomes and should be seen as priorities and delivered through programmes of complex interventions by specialist staff integrated within the same service. Restraint and seclusion should be avoided wherever possible and all staff should have adequate training in the use of early de-escalation of violence. Adequate staff training in appropriate clinical skills and regular supervision should be provided and this should support service user involvement in decision making and positive therapeutic relationships between staff and service users. There should be clear lines of clinical governance that ensure adherence to evidence-based guidelines and attention should be paid to service users' physical health through regular screening.

### The Strength of the Evidence

We have deliberately undertaken a broad systematic review in order to inform a whole systems approach to the care of long term mental illness. Although we can be criticised for being too broad, it is only when the full scope of service provision is seen together that a desirable balance of various therapies and services becomes clearer.

Evidence was strongest for specific interventions for the treatment of schizophrenia, especially CBT, family psychoeducation and supported employment. Psychoeducation and CBT were found to promote service users' knowledge about their mental health problems and their self-management of symptoms, and family psychoeducation improved service users' support networks. Supported employment, particularly IPS, which has been further evidenced by the recently published EQOLISE Study [129], improves service users' chances of gaining competitive employment and brings other benefits economically, socially and in terms of citizenship and self-esteem.

Through our assessment measure we aimed to present the quality of each study. These scores were used in determining how much weight to give to any one study's findings. However, the assessment of quality also has its limits. Several studies achieved the maximum possible score. This was mostly found among the quantitative studies. This does not necessarily infer that these studies were without limitation - it simply denotes that the study met the requirements we laid out. Furthermore, studies which may have been very well designed and conducted could not receive full marks if they were conducted in only one centre. However, the inclusion of a number of sites as a quality criterion seems reasonable since multi-centre studies provide greater evidence than single site studies for the generalisability of results. In addition, this is in keeping with the Medical Research Council's accepted hierarchy of evidence [16].

We noted a lack of studies of high quality in relation to other specific interventions (such as cognitive remediation, arts therapies, compliance therapy, relapse prevention, coping skills training), the living environment and staff training and support. Lower quality studies (with an average quality rating of less than 10) were found in relation to living environment, integrated therapy, physical health, restraint and seclusion, the therapeutic relationship and service user involvement. Fewer and poorer quality studies were found in relation to interventions for comorbid substance misuse, occupational therapy, autonomy and clinical governance.

The lack of evidence found for certain components of care may be because specific interventions lend themselves more easily to effectiveness research than other aspects of care such as therapeutic relationship and clinical governance. That limitation does not, however, explain the weak evidence base for quite specific treatments such as social skills therapy, arts therapies, occupational therapy, compliance therapy, supportive therapy and coping skills training. However, we cannot say that several of the interventions we do not regard as key to patients' outcomes may not be shown to be useful given more research. It may be simply that funding bodies for research do not regard them as a priority. Certainly many of these interventions (e.g. occupational therapy) are highly valued by mental health professionals and usually provided in these institutions.

The majority of studies were from the USA and UK, which provides a further limitation as findings may not be generalisable to other cultures and service settings. However, the systematic nature of our approach means that the papers included represent the best available evidence relevant to components of institutional care for people with longer term mental health problems.

It is unsurprising that a positive therapeutic alliance between staff and service users is associated with better outcomes. This finding highlights the importance of providing a collaborative, client-centred approach where treatments and interventions are discussed and negotiated, non-coercively, between professionals and service users and where individualised care is planned together and reviewed. However, for clients who are more functionally and cognitively impaired such that participation in their care may not be possible, Frese et al [130] suggest that evidence-based interventions should be used and more collaborative approaches implemented as the client's mental state and functioning improve.

### Relationship with Recovery

Much of the evidence provided overlaps with the concept of "Recovery", which originated from the narratives of people with personal experience of mental health problems [131], and recovery-orientated services. The most widely used definition of Recovery is given by Anthony [132]: "A deeply personal, unique process of changing one's attitudes, values, feelings, goals, skills and roles. It is a way of living a satisfying, hopeful and contributing life even with the limitations caused by illness." Four domains have been identified that encompass the concept: empowerment; hope and optimism; knowledge about mental illness and available treatments; and satisfaction with quality of life [133]. Markers of Recovery include: working, studying and participating in leisure activities in mainstream settings; having good family relationships; living independently; having control of one's self-care, medication and money; having a rewarding social life; taking part in the local community; voting; and achieving greater satisfaction with life [134-136].

Mueser et al [137] describe how the approaches to illness management in traditional rehabilitation programmes can facilitate Recovery (e.g. by assisting the individual to gain mastery over their symptoms, formulate and achieve goals, and improve their self-efficacy and self-esteem) and incorporate key aspects of Recovery-orientated services such as collaborative working between clinicians and patients.

Our review strongly supports the view that community and hospital based facilities for individuals with severe mental health problems should deliver "Recovery-oriented" services and many of the existing approaches and interventions already deliver aspects of this. For example, providing care in settings that are least restrictive and provide a homely and personalised environment, ideally in the community, appears to facilitate service users' community integration and autonomy. Facilities that provide service users with opportunities for autonomy have greater success in assisting their clients to gain skills and confidence as well as greater client satisfaction [138]. In addition to collaboration in their care, service user involvement in developing, running and reviewing services has been shown to have positive outcomes for both the services and the service users who take on these roles [117,118]. The American Association of Community Psychiatrists' Guidelines for Recovery Orientated Services [124], which were developed from clinical consensus and literature review, include many of the evidence-based interventions identified in this review and highlight the need for services to include service users in organisations' planning of services and their clinical governance processes.

## Conclusion

Our synthesis of the literature has examined of the strength of the evidence for various components of institutional care for people with longer term mental health problems. An "ideal" institution should be small and community based and maximise flexibility, privacy, engagement and positive therapeutic relationships. It should provide regular physical health screening and specific interventions (CBT, family interventions involving psychoeducation, and supported employment) through integrated programmes for service users with a diagnosis of schizophrenia. Restraint and seclusion should be avoided whenever possible and staff should have adequate training in de-escalation techniques. Regular staff supervision should be provided with clear lines of clinical governance that ensure adherence to evidence-based guidelines.

## Competing interests

The authors declare that they have no competing interests.

## Authors' contributions

TT carried out the electronic search, screened all relevant abstracts for eligibility, assessed included papers for quality, extracted data, allocated papers to domains and drafted the manuscript. HK conceived of the study, participated in its design, screened all relevant abstracts for eligibility, assessed included papers for quality, agreed upon key domains, ensured reliability of paper allocation and helped to draft the manuscript. MK participated in the study design and helped to draft the manuscript. CW, PT and SW participated in the study design. All authors read and approved the final manuscript.

## Pre-publication history

The pre-publication history for this paper can be accessed here:



## Supplementary Material

Additional file 1 **Excluded studies**. Excluded studies with reason for exclusion. Click here for file
